# Supplementary material for: Polymorphisms in Pfkelch13 domains before and after the introduction of artemisinin-based combination therapy in Southwest Nigeria
Source: PLoS One. 2025 Mar 31;20(3):e0316479. doi: 10.1371/journal.pone.0316479 (PMC11957316; doi:10.1371/journal.pone.0316479)

Samples: 13822
Bases: 833
Average spacing: 17

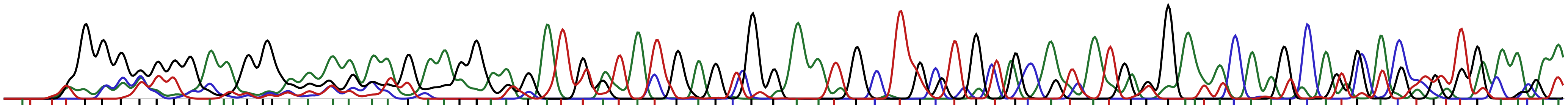

10 20 30 40 50 60 70 80

AT T T G G G G G G G G A A A G G G A A A A A A G A A G G A A G A T T A T A T G A G C G G A A T G C T G C T G T G C A T A T G G G A A T A C A A G C A T G A C T G T T G A A C A A

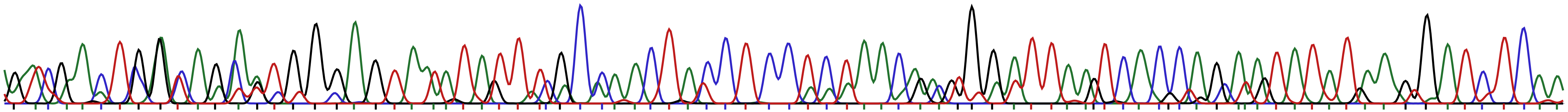

90 100 110 120 130 140 150 160 170

G A C A A C T G G T A G A G T G G G A G T A A A T A T T T C G C C A A C T A C C T C C T C T A A C A A G G G A T T A A G T C A C G C A G A A A T A T A T A A G G A T C T C A A

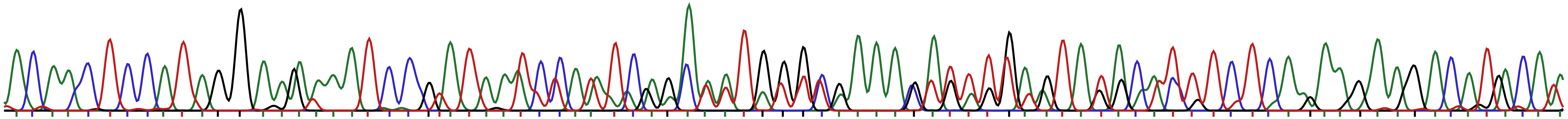

180 190 200 210 220 230 240 250 260

A C A A C T C C A T A G G A A A A A A T C C G T A T A A T C C A A T C A G A A A T G G G C A A A A G A T T T G A A T A T A C A T T T C T C A G A A G A A G A C A T A C A A

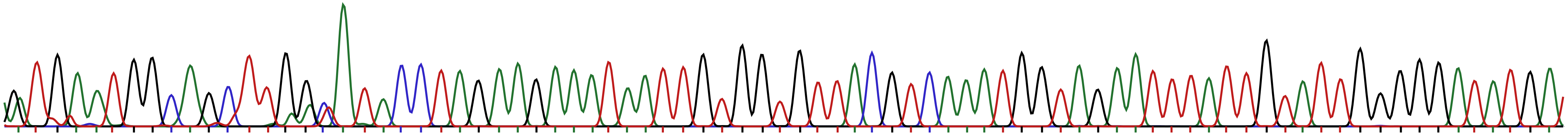

270 280 290 300 310 320 330 340

A T G A A T G G C A G C T T G G C A T A C C T A G A A G A A A T A A T T G T G G T G T T A C G T C A A A T G G T A G A A T T T A T T G T A T T G G G G G A T A T G A

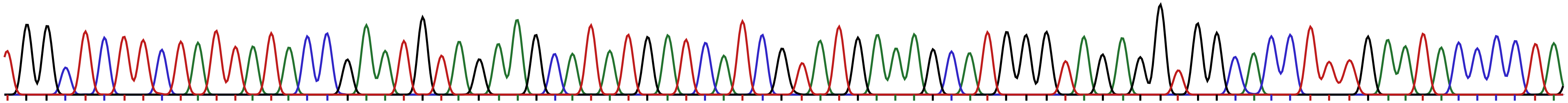

350 360 370 380 390 400 410 420

T G G C T C T T C T A T T A T A C C G A A T G T A G A A G C A T A T G A T C A T C G T A T G A A A G C A T G G G T A G A G G T G G C A C C T T T G A A T A C C C C T A

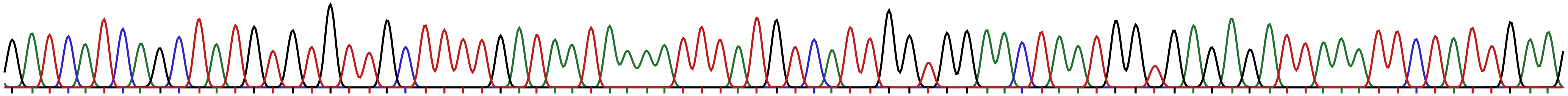

430 440 450 460 470 480 490 500

G A T C A T C A G C T A T G T G T G T T G C T T T T G A T A A T A A A A T T T A T G T C A T T G G T G G A A C T A A T G G T G A G A G A T T A A A T T C T A T T G A A

Samples:	13822	Page: 2 / 3
Bases:	833	8/17/2022
Average spacing:	17	

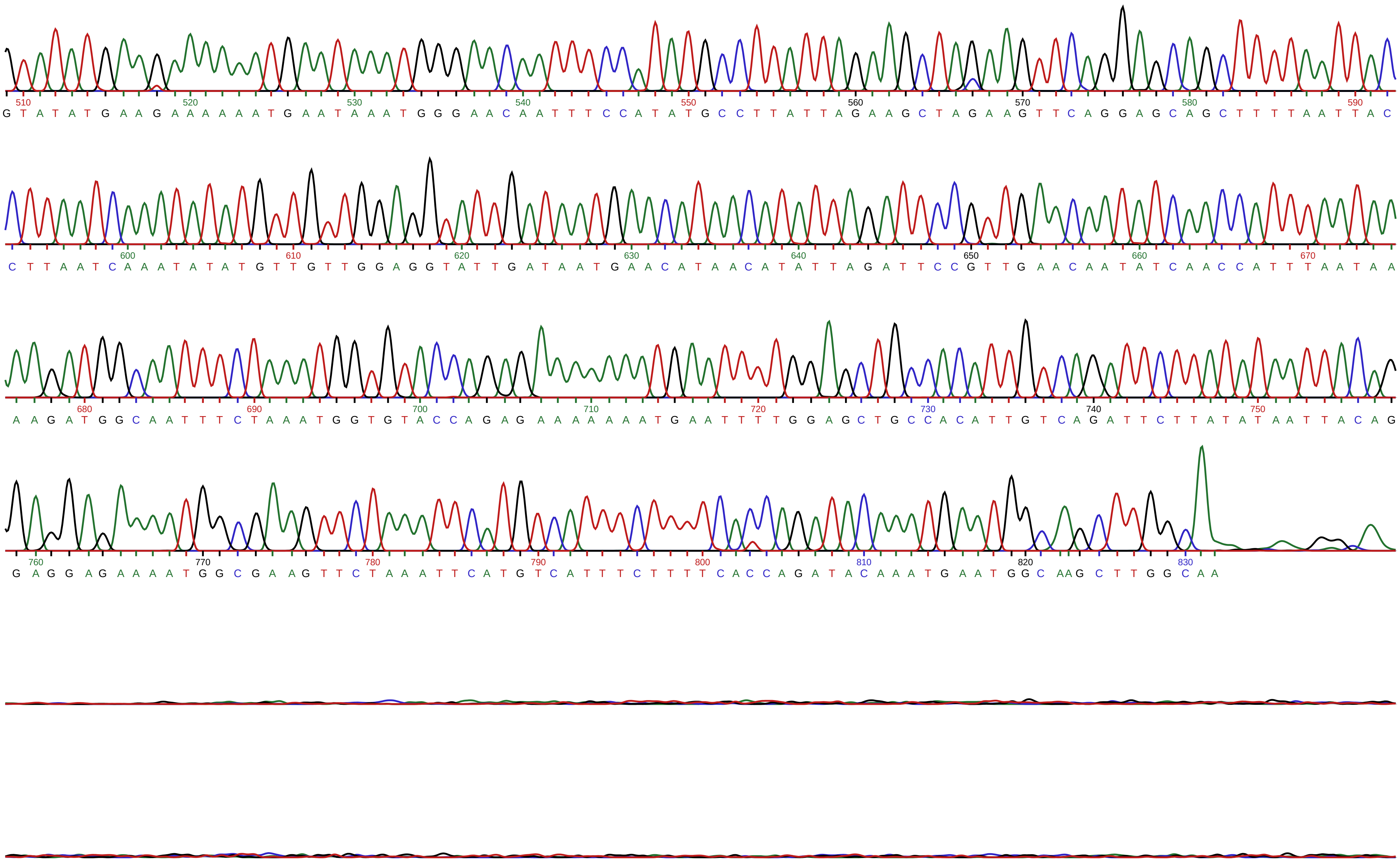

Samples: 13822
Bases: 833
Average spacing: 17

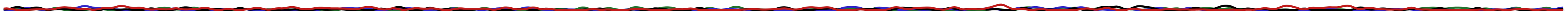

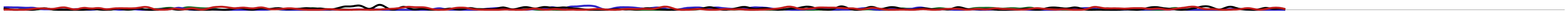

Supplement: Supporting information 2 — (ZIP) [file pone.0316479.s002.zip › 201KN1F_PREMIX_Plate_KELCH1_A08.pdf]
